# Insights into molecular and metabolic events associated with fruit response to post-harvest fungal pathogens

**DOI:** 10.3389/fpls.2015.00889

**Published:** 2015-10-20

**Authors:** Noam Alkan, Ana M. Fortes

**Affiliations:** ^1^Department of Postharvest Science of Fresh Produce, Volcani Center, Agricultural Research OrganizationBet Dagan, Israel; ^2^Biosystems & Integrative Sciences Institute, Faculdade de Ciências de Lisboa, Universidade de LisboaLisboa, Portugal

**Keywords:** post-harvest, ripening, plant response, phytohormones, cuticle, softening, phytoalexin, quiescent

## Abstract

Due to post-harvest losses more than 30% of harvested fruits will not reach the consumers’ plate. Fungal pathogens play a key role in those losses, as they cause most of the fruit rots and the customer complaints. Many of the fungal pathogens are already present in the unripe fruit but remain quiescent during fruit growth until a particular phase of fruit ripening and senescence. The pathogens sense the developmental change and switch into the devastating necrotrophic life style that causes fruit rotting. Colonization of unripe fruit by the fungus initiates defensive responses that limit fungal growth and development. However, during fruit ripening several physiological processes occur that correlate with increased fruit susceptibility. In contrast to plant defenses in unripe fruit, the defense posture of ripe fruit entails a different subset of defense responses that will end with fruit rotting and losses. This review will focus on several aspects of molecular and metabolic events associated with fleshy fruit responses induced by post-harvest fungal pathogens during fruit ripening.

## Introduction

**FIGURE 1:**
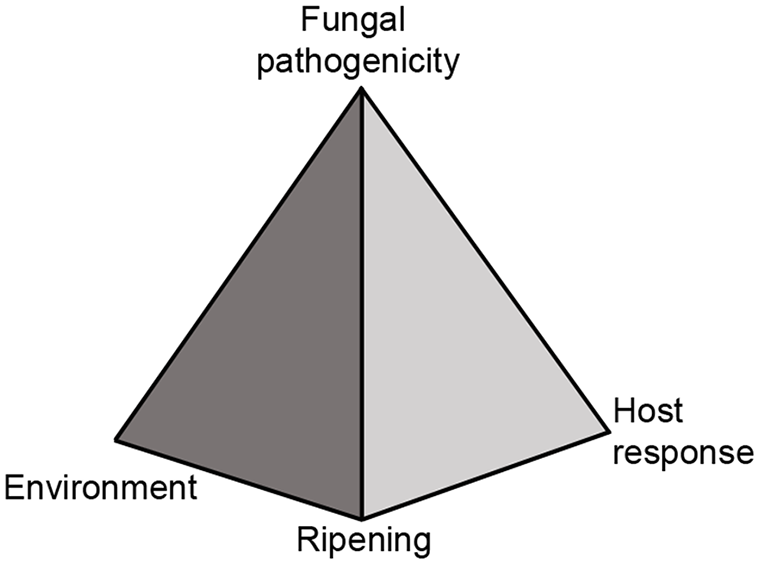
**Disease pyramid of post-harvest fruits and pathogenic fungi interaction.** The disease of post-harvest fruits and pathogenic fungi is regulated by four elements: (1) Fungal pathogenicity, (2) host response, (3) environment, and (4) ripening or senescence.

Food waste from the grower to the consumer is an important issue as it depletes natural resources. Recent researches and surveys done by NRDC (Natural Resources Defense Council), USDA (US Department of Agriculture), FAO (Food and Agriculture Organization of the United Nations), and the OECD (Organization for Economic Co-operation and Development) revealed that food losses are estimated to be more than 33% (Gustavsson et al., 2011; Lipinski et al., 2013; Buzby et al., 2014; Okawa, 2014). Post-harvest losses of fruits and vegetables are even higher and are estimated to be 40–50%. Post-harvest fruit rotting are a major cause of those losses and are chiefly caused by fungal pathogens after fruit ripening. In a manner similar to foliar diseases that occur in the field, several factors as: fungal pathogenicity, host response and environment determine the outcome of host resistance or susceptibility. However, in post-harvest diseases fruit ripening is another major component that will determine fruit resistance and must be considered (**Figure 1**).

### Post-harvest Disease Development

Fruits infected by post-harvest fungal pathogens develop, in general, disease symptoms after harvest and during storage. Post-harvest fungal pathogens germinate and enter the fruit by breaching the host cuticle (Emery et al., 2000; Alkan et al., 2015). This is achieved by: degrading host cuticle (Rijkenberg et al., 1980), entering through natural openings of the host and wounds (Barkai-Golan, 2001), or by living endophytically in the stem end (Johnson et al., 1992; Prusky et al., 2009). When those particularly insidious pathogens encounter unripe fruit these fungi often remain quiescent and confined to the initial site of introduction. They are unnoticed to visual examination, for as long as months of storage, until the harvested fruit ripen (Prusky et al., 2013). Several species of fungal pathogens, such as *Colletotrichum, Alternaria, Botrytis, Monilinia, Lasiodiplodia, Phomopsis*, and *Botryosphaeria* have been reported to live quiescently in their hosts until the fruits ripen (Prusky et al., 1981; Adaskaveg et al., 2000; Prins et al., 2000). As fruit ripen, post-harvest fungal pathogens switch to aggressive growth. At this aggressive stage, the fungi are necrotrophs, which kill the host cell and obtain nutrients from the host, leading to decomposed fruit tissue and decay (Prusky, 1996; Prusky et al., 2013). However, before this devastating stage those fungi adopt different types of life styles. Some fungi as *Lasiodiplodia, Phomopsis, Colletotrichum, Alternaria* and others, cause stem-end-rot and colonize the stem-end by adopting endophytic-like lifestyle before fruit ripening (Johnson et al., 1992). Other fungi, e.g., *Colletotrichum* are defined as hemibiotroph, those fungi live quiescently as biotrophs in unripe fruit cells without killing them (O’Connell et al., 2012; Alkan et al., 2015). In a parallel manner, fully necrotrophic fungi as *Botrytis* can infect and live in a restricted 1–3 cells of unripe fruit without damaging the surrounding tissue (Cantu et al., 2008a).

### *Botrytis* and *Colletotrichum* Model

Due to lack of omics data and in-depth knowledge in the stem-end-rot pathosystems, this review will focus on the better understood *Colletotrichum* (anthracnose) representing hemibiotrophic fungi and on *Botrytis* (gray mold) as necrotrophic fungi. These fungi are two of the most common post-harvest fruit disease agents that are known to attack many economically important fruits and present problems world-wide (Sutton, 1992; Cannon et al., 2000; Hyde et al., 2009). *Colletotrichum gloeosporioides* causes the anthracnose disease to at least 470 host genera (Sutton, 1980; Hyde et al., 2009) and *Botrytis cinerea* causes the gray mold disease on over 200 species of fruit. On unripe fruit, *Colletotrichum* conidia germinate and develop appressoria which penetrate the fruit cuticle via an infection peg. *C. gloeosporioides* enters the quiescent stage whereupon two distinct structures develop: dendritic-like protrusions which form within the fruit cuticle and swollen hyphae which colonize the first epidermal cell layer but advance no further (Alkan et al., 2015). When *C. gloeosporioides* germinates on the cuticle of ripe fruit it germinates as on green fruit and goes through a short biotrophic stage. Only this time it is much more rapid and the quiescent structures immediately switch to necrotrophic growth. This indicates that hemibiotrophic growth in *C. gloeosporioides* is developmentally cued when encounter with fruit cuticle. On the other hand, *Botrytis* spore germlings tend to penetrate through small wounds or cracks in the epidermis or cuticle of unripe fruit and remain confined within the lumen of the wounds (Williamson et al., 2007; Cantu et al., 2008a). When the hemibiotrophic *C. gloeosporioides* germinates on small wounds of unripe fruits, its colonization skips the biotrophic-like stage and it adopts the necrotrophic strategy, similarly to *B. cinerea* (Alkan et al., 2015). Growth of either pathogen on wounds in unripe fruit is limited for long periods, and upon ripening, both pathogens become necrotrophic, degrade host tissues and produce symptoms of disease (Prusky, 1996; Prusky et al., 2013).

### Unripe Fruit Tolerance and Changes Occurring during Ripening

**FIGURE 2:**
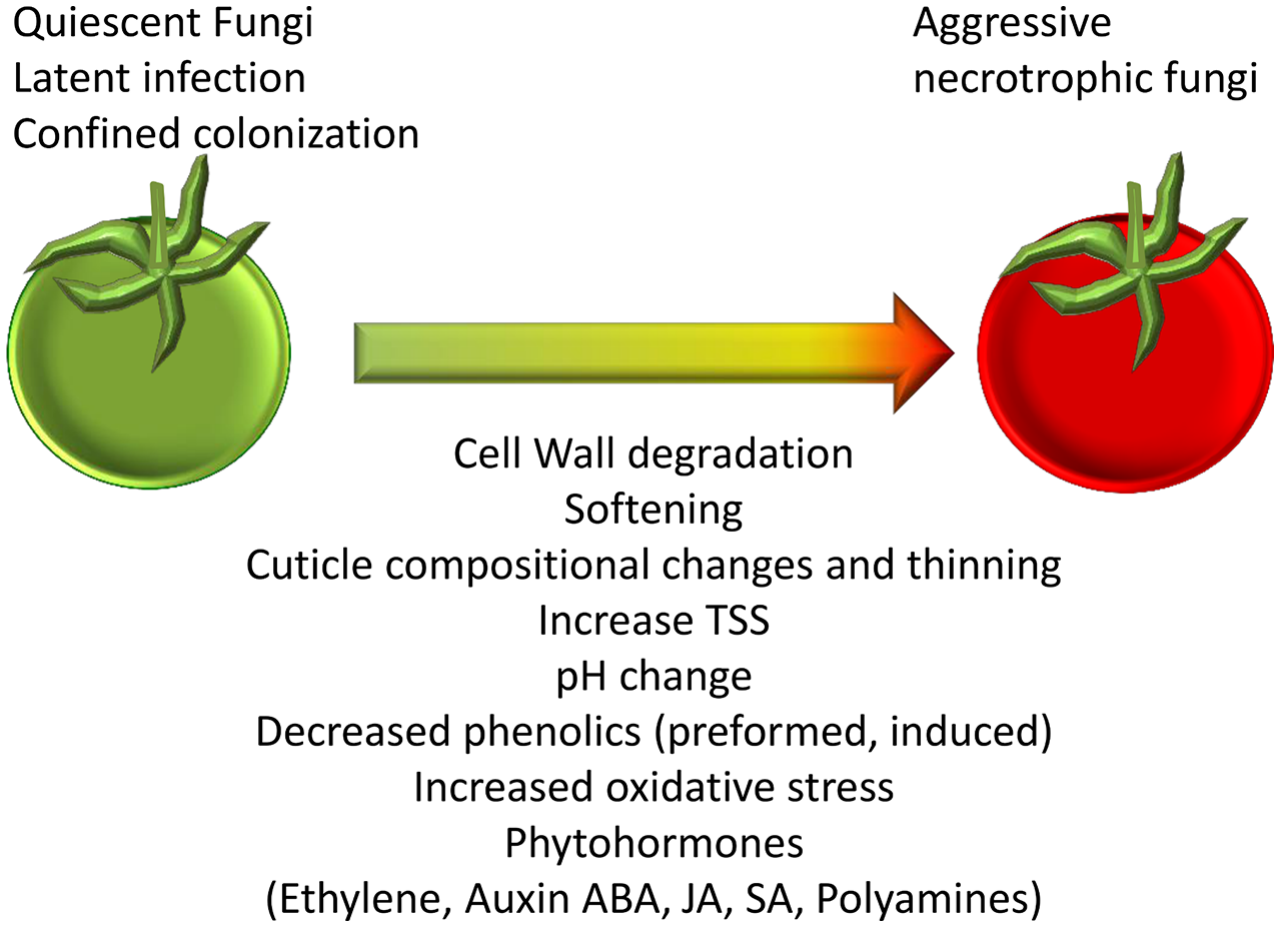
**Changes in the fruit that occur during ripening and senescence that regulate susceptibility.** Morphological and biochemical changes taking place during the transition from unripe and tolerant to ripe and susceptible fruit. These changes could stimulate the release from quiescent/ latent or confined colonization to the aggressive colonization of the necrotrophic fungi in ripe fruits.

During fruit ripening, significant physiological shifts occur: cell wall remodeling (Brummell et al., 1999; Huckelhoven, 2007), soluble sugar accumulation, decrease in the amount of phytoanticipins and phytoalexins (Prusky, 1996); decline of inducible host defense responses (Beno-Moualem and Prusky, 2000); cuticle biosynthesis (Bargel and Neinhuis, 2005) and changes in the ambient host pH (Prusky et al., 2013; **Figure 2**). Most of those changes are thought to be governed by complex hormonal signals including ethylene, ABA, jasmonic acid (JA), and salicylic acid (SA), which occur during natural fruit ripening (Giovannoni, 2001; Seymour et al., 2013). Interestingly, similar phytohormones are regulated in the host in response to pathogens (Blanco-Ulate et al., 2013; Alkan et al., 2015). In response to the changes in the host, pathogens alter the enzymes and compounds they produce which allow them to infect and break down or macerate the fruit tissue (Blanco-Ulate et al., 2014; Agudelo-Romero et al., 2015; Alkan et al., 2015). Signals for release from quiescence probably occur during fruit ripening and may include: disassembled cell wall substrates, alterations in cuticle and other signals (Cantu et al., 2008a,b; Mengiste et al., 2012; **Figure 2**). When the fungi are re-activated they induce rotting disease that impairs crop quantity, quality and appearance. These aspects will be discussed in the following sections.

## Host Factors Modulating Post-Harvest Fungal Development

Recently, with the expansion of omics technique several observations elaborated on the involvement of differential response of ripe and unripe fruit to fungal pathogens (Blanco-Ulate et al., 2014; Agudelo-Romero et al., 2015; Alkan et al., 2015).

This review will describe both the changes occurring during fruit ripening and the fruit response to post-harvest fungal pathogens.

### Phytohormones: Jasmonate-salicylate Crosstalk and More

Phytohormones are well-known to affect fruit ripening (Burg and Burg, 1965; Alexander and Grierson, 2002) and the defense responses to pathogens (Alkan et al., 2012, 2015; Blanco-Ulate et al., 2013; Agudelo-Romero et al., 2015). Important signaling roles have been ascribed to classical defense hormones SA, JA, abscisic acid (ABA), and ethylene (ET) in molding plant–pathogen interactions (Fujita et al., 2006; Spoel and Dong, 2008). Gibberellic acid (GA), auxin (IAA), brassinosteroids (BRs), and cytokinines (CK) have recently been emerged as important modulators of plant defenses against microorganisms based mostly on vegetative tissues data and on the lifestyle of the infecting pathogen (Robert-Seilaniantz et al., 2011).

#### Jasmonate-salicylate Crosstalk

**FIGURE 3:**
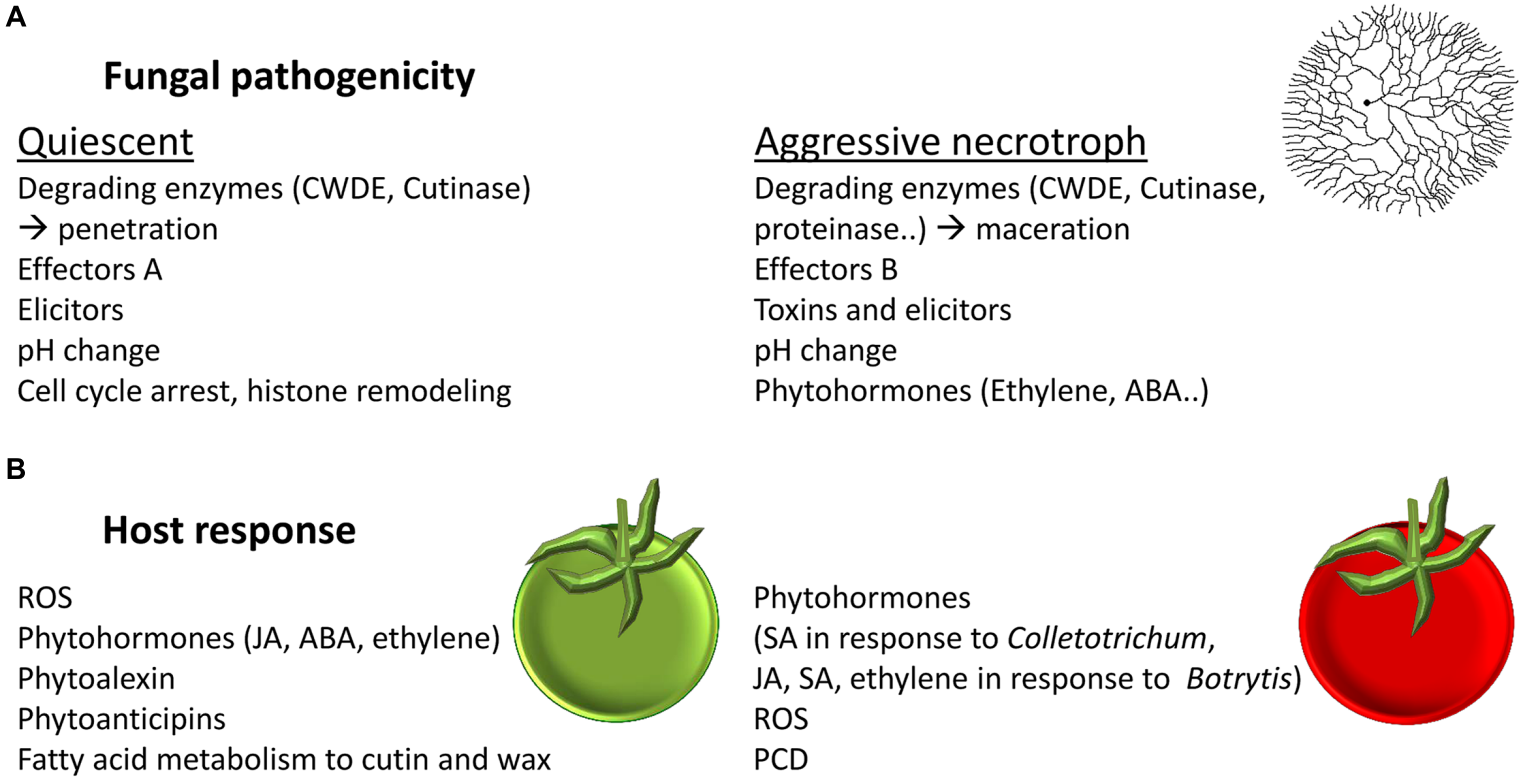
**Fungal pathogenicity and host response at different stages of fruit maturity. (A)** Fungal pathogenicity factors during quiescent and during aggressive necrotrophic stage. **(B)** Host response to fungal colonization in unripe and in ripe fruit.

**FIGURE 4:**
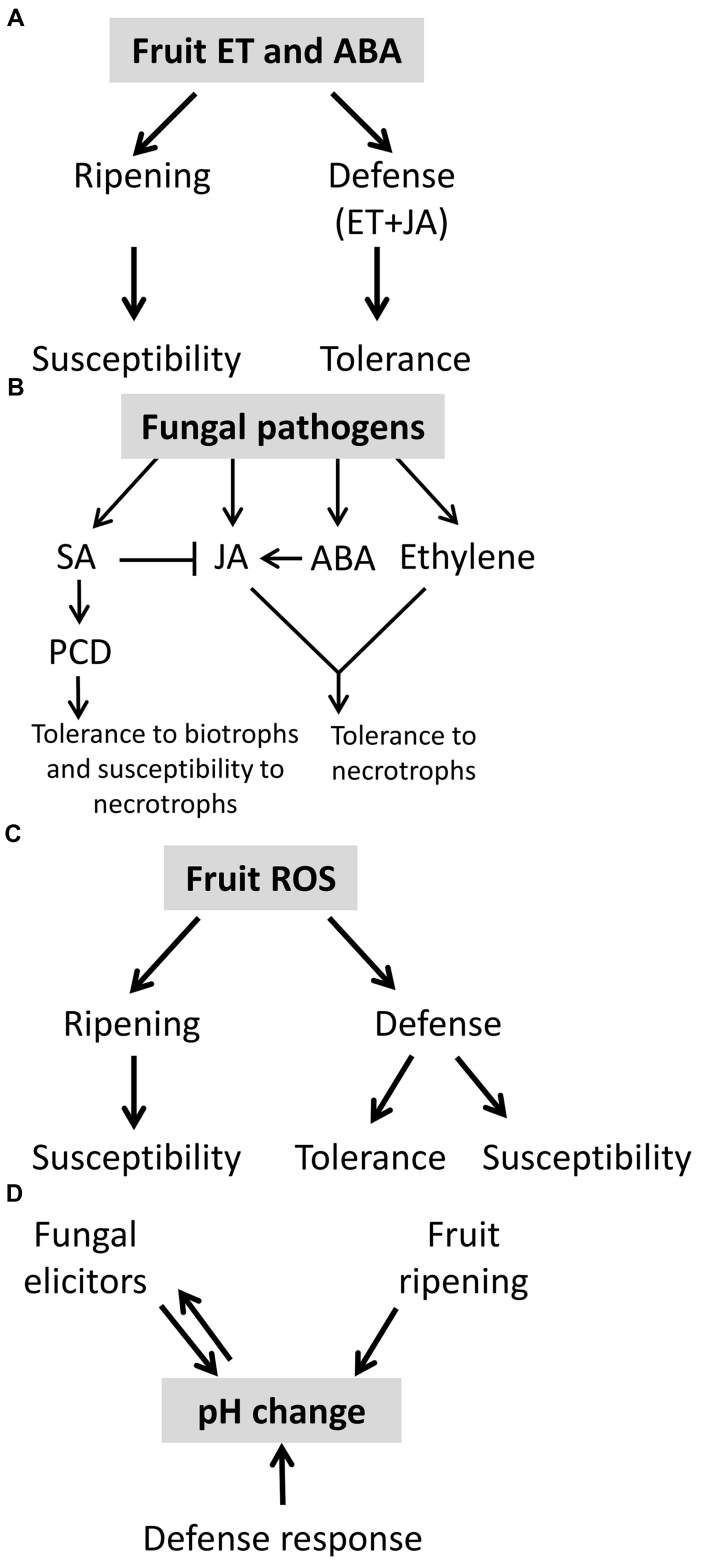
**Role of phytohormones, ROS, and pH in post-harvest disease. (A)** Ethylene and abscisic acid (ABA) activate fruit ripening and susceptibility in fruit but also participate in the defense response to pathogens together with JA. This interplay of hormonal signals can lead to increased tolerance. **(B)** Fruit defense response to fungal pathogens is mediated by phytohormones as salicylic acid (SA), jasmonic acid (JA), ABA, and ethylene. Phytohormones cross-talk can determine fruit tolerance to biotrophic or necrotrophic fungal pathogens. JA and ethylene are classically reported to be involved in tolerance to necrotrophs and SA to biotrophs. **(C)** ROS is one of the components that control fruit ripening and thus susceptibility. ROS also participate in fruit defense response, depending on it relative concentration it could lead to susceptive or tolerant response. **(D)** Fruit ripening, fruit defense response and fungal elicitors modify the pH in the court of host-pathogen interaction.

Salicylic acid and JA signaling pathways are generally considered as antagonistic dependent on NPR1 and hormone concentration (Spoel et al., 2007; Spoel and Dong, 2008; Pieterse et al., 2012). This interplay between SA and JA was suggested to optimize host-response to pathogen’s lifestyle (Glazebrook, 2005; Spoel et al., 2007; Spoel and Dong, 2008; Pieterse et al., 2012). In vegetative tissue it is commonly postulated that an effective responses to biotrophic pathogens are typically mediated by SA and programmed cell death (PCD; Glazebrook, 2005; Spoel et al., 2007), and responses to necrotrophic pathogens, which benefit from host cell death, involve JA and ethylene signaling (Glazebrook, 2005; Spoel et al., 2007; **Figures 3** and **4B**).

During normal fruit ripening many phytohormones as ethylene, IAA, ABA, GA, JA, and SA are regulated (Bari and Jones, 2009; Symons et al., 2012; Zaharah et al., 2012; Seymour et al., 2013; **Figure 2**), which complicate the effective fruit response to pathogens. Indeed, in infections of grapes and tomato fruit with *Botrytis* (Blanco-Ulate et al., 2013; Agudelo-Romero et al., 2015) and tomato fruit infections with *Colletotrichum* (Alkan et al., 2015) several stress hormone responsive pathways including ethylene, ABA, JA, and SA were regulated. In fruit, high levels of ET and ABA, which stimulate senescence/ripening processes, may facilitate colonization by necrotrophs. The balance between SA and JA responses seems to be crucial for fruit resistance (Blanco-Ulate et al., 2013; Alkan et al., 2015).

During the biotrophic-like quiescence stage of *C. gloeosporioides* the resistant unripe fruit mainly responded through activation of JA, ethylene, and ABA (Alkan et al., 2015). However, the ripe tomato is more susceptible to disease and the fungi adopt a necrotrophic mode of host colonization. In the fruit response to necrotrophic infections, SA biosynthetic, signaling and response pathways were activated (Alkan et al., 2015; **Figure 3**). SA signaling was shown to have an important role in necrotrophic colonization stage of *Colletotrichum* on tomato fruit by inducing cell death and likely as a means to suppress JA mediated defense response (Alkan et al., 2012). The activation of this pathway was dependent on NADPH oxidase activity that was induced by ammonia secreted by the fungus (Alkan et al., 2009). Indeed, JA application leads to increase tolerance and SA application leads to PCD and increased susceptibility to *C. gloeosporioides* at it necrotrophic stage (Alkan et al., 2012). Similarly, ripe *NahG* tomato fruit mutant, lacking SA responses, showed increased tolerance to *C. gloeosporioides.* In a reciprocal manner, the *Spr1* mutant, deficient in JA signaling, showed increased susceptibility (Alkan et al., 2015).

The responses of tomato fruit to *Botrytis* involved several stress hormone responsive pathways including ethylene, ABA, JA, and SA in a complex manner that differ during infections of ripe and unripe tomato fruit. In general, both grape and tomato defense response to *Botrytis* were mainly mediated by JA and ET (Blanco-Ulate et al., 2013; Agudelo-Romero et al., 2015; **Figure 3**). Similarly, *Arabidopsis* leaves response to *Botrytis* was mediated mainly by JA and ET (AbuQamar et al., 2006). However, in unripe tomato fruit the *NahG* gene showed susceptibility to *Botrytis* (Blanco-Ulate et al., 2013). This result suggests that SA has an important role in unripe fruit resistance to *Botrytis*. In grapes of susceptible cultivar infected with *B. cinerea*, the pathogen causes shutdown of defenses, which are regulated by SA and are expressed during the normal ripening (Agudelo-Romero et al., 2015). It remains to be established whether SA-mediated defense system is inhibited or not in resistant cultivars.

In climacteric fruit ET and ABA stimulate ripening and may affect the host defense response (Seymour et al., 2013). Thus, resistance of *sitiens* mutants at ripe fruit stage to *Botrytis* and ABA elevation in response to *Botrytis* was correlated to susceptibility (Blanco-Ulate et al., 2013). The ethylene and ABA elevation seems to play a dual role, on the one hand it correlated with JA and ethylene resistance response, and on the other hand it induced ripening and increase susceptibility (**Figure 4A**). This assumption will be further discussed below.

#### Not Only SA and JA Crosstalk

As discussed above, major responses to fungal pathogens are mediated by ethylene, ABA, JA, and SA phytohormones (**Figure 3**). Additional phytohormones and growth regulators such as gibberellin, cytokinins, steroid, polyamines, and BRs were reported to affect fruit response to fungal pathogens. For example gibberellin-treated persimmon fruit had increased resistance of to *Alternaria alternata* by delaying fruit maturation and reducing cuticle cracks (Eshel et al., 2000; Biton et al., 2014). Gibberellin seems to act also in an alternative way, the DELLA transcription factors enable plants to respond to gibberellin; this mechanism seems to maintain transient growth arrest and lead to plant response to biotic and abiotic stress (Harberd et al., 2009). DELLA transcription factors were shown to promote susceptibility to biotrophs and resistance to necrotrophs in leaves (Navarro et al., 2008).

Polyamines may have a role in response to post-harvest pathogens. In fact, genes involved in polyamine biosynthesis are up-regulated in *Botrytis-*grapes pathosystem (Geny et al., 2003; Agudelo-Romero et al., 2015). Over-expression of *ARGAH2* in *Arabidopsis* leads to enhanced resistance to *B. cinerea*, thus suggesting a role for the polyamine arginase in plant resistance (Brauc et al., 2012). Furthermore, polyamines seem to be co-regulated with ethylene biosynthesis and seem to co-work with ABA (Bitrian et al., 2012).

Steroids such as β-sitosterol and stigmasterol have been previously shown to be involved in plant–pathogen interactions (Griebel and Zeier, 2010). Indeed, several genes involved in steroid biosynthesis were also upregulated in grape and tomato response to *Botrytis* (Blanco-Ulate et al., 2013; Agudelo-Romero et al., 2015). Furthermore, BRs play a role in grape ripening (Symons et al., 2006) and are known to change in plants response pathogen infection (Krishna, 2003). BAK1, known for BRs signaling, contributes to pathogen associated molecular pattern-triggered immunity (PAMP) to necrotrophic pathogens (Mengiste et al., 2012). Loss of BAK1 results in increased susceptibility to the necrotrophic fungi (Kemmerling et al., 2007). Several genes coding for BAK1 were down-regulated in grapes infected with *Botrytis* at the onset of ripening (Blanco-Ulate et al., 2013; Agudelo-Romero et al., 2015). Application of BRs reduced *Penicillium expansum* decay in jujube fruit and delayed fruit senescence (Zhu et al., 2010).

#### Ethylene Dual Role in Ripening and Defense Response

Ethylene is a key post-harvest hormone with a dual role. In climacteric fruit ripening (Giovannoni, 2001; Seymour et al., 2013), the presence of ethylene will lead to susceptibility, however, ethylene could also acts as a defense hormone together with JA (Spoel and Dong, 2008; **Figure 4A**).

In tomato, a climacteric fruit, ethylene is known to commence during the breaker stage of fruit ripening (Seymour et al., 2013). Post-harvest fungal pathogen as *B. cinerea* and *C. gloeosporioides* infections induce the ethylene biosynthesis pathway, transcription factors as non-ripening (*NOR*), ripening-inhibitor *(RIN*) and never-ripe *(NR*) and the ethylene-regulated defense genes in both ripe and unripe tomato fruit (Blanco-Ulate et al., 2013; Alkan et al., 2015). Therefore, they enhance the ripening process and hasten the release from quiescence.

The increased susceptibility of ripe fruit to *B. cinerea* depends on the ripening regulator *NOR*, but not on *RIN*, and only partially on the fruit’s perception of ethylene. The *rin* mutant fruits and those treated with 1-MCP did not ripe but, nevertheless, were susceptible to *B. cinerea* (Cantu et al., 2009; Blanco-Ulate et al., 2013). The differential effect of *NOR* and *RIN* indicate the existence of a specific susceptibility factor. Although *nor* and *rin* act together in a cascade for ripening *nor* appears to have a more global effect indicating that it operates upstream of rin (Osorio et al., 2011). Inspection of the differential expression of genes in these mutants may point to a source for susceptibility.

Applications of 1-MCP, an inhibitor of ethylene perception, are widely used due to the beneficial effects in delaying fruit senescence and prolonging storage (Watkins, 2006; Watkins and Nock, 2008). Indeed, in apples (Saftner et al., 2003), peaches (Liu et al., 2005), and plums (Menniti et al., 2004) 1-MCP treatment reduced fungal pathogens rotting. However, in tomato (Su and Gubler, 2012; Biswas et al., 2014), avocado (Woolf et al., 2005), custard apple, mango, and papaya (Hofman et al., 2001), citrus (Porat et al., 1999), apples (Janisiewicz et al., 2003) 1-MCP promoted susceptibility to pathogens. It seems that 1-MCP could affect citrus, strawberry, and tomato fruits susceptibility in a concentration dependent manner. At low concentrations it enhances tolerance, but reduces it at high concentrations (Ku et al., 1999; Dou et al., 2005; Blanco-Ulate et al., 2013). Thus, small amounts of endogenous ethylene may be necessary to maintain basic levels of resistance to pathogens due to ethylene involvement in regulation of plant defense genes (Marcos et al., 2005).

These results support the conclusion that ethylene has dual opposing roles: as a ripening hormone it promotes susceptibility (Burg and Burg, 1965) and as a participant in hormone-activated defense responses ethylene provide resistance (Glazebrook, 2005; Spoel et al., 2007). Thus, the timing of ethylene release, perception and the levels of ethylene are likely to be crucial for the outcomes of resistance or susceptibility (**Figure 4A**).

### ROS Role in Ripening and Defense Response

Accumulation of reactive oxygen species (ROS) are the result of the balance between ROS production and antioxidant activity. The mitochondria chloroplast and peroxisome are all potential sources for ROS production. ROS and particularly hydrogen peroxide (H_2_O_2_) and singlet oxygen (^1^O_2_) contribute to fruit ripening and senescence (Brennan and Frenkel, 1977; Lacan and Baccou, 1998; Rogiers et al., 1998; Tian et al., 2013). In grape and tomato, ROS, lipid peroxidation, and protein oxidation were increased at breaker stage (**Figure 4C**). In many fruits, storage is associated with an increase in ROS, which results either from increased ROS production or from a decrease in antioxidative makeup (Hodges, 2003). Antioxidants inhibit fruit ripening and senescence (Lester, 2003), while, high O_2_ or application of H_2_O_2_ leads to increase ROS and senescence (Tian et al., 2013). Oxidative damage of several mitochondrial proteins, which involved in fruit senescence would result in impairment of mitochondrial function and lead to fruit senescence (Tian et al., 2013).

Many post-harvest fungi can modulate host ROS production. Fungi secrete elicitors, toxins and antioxidants in order to modify the plant ROS production (Aver’Yanov et al., 2012; Alkan et al., 2013a). For example, oxalate secreted by *Sclerotinia* generates reducing conditions and inhibited plant oxidative burst at pH 3–4 (Cessna et al., 2000; Williams et al., 2011). In contrast, oxalic acid also induced plant NADPH oxidase and ROS production that correlated with a pH increase to 5–6, that later induced host PCD (Kim et al., 2008). Another example is ammonia that is secreted by *Colletotrichum*, which activated the tomato NADPH oxidase, this resulted in oxidative burst leading to induction of SA mediated defense response, host cell death and enhanced necrotrophic colonization (Alkan et al., 2009, 2012). How these small molecules manipulate the NADPH oxidase is unknown.

Plant ROS can be toxic, e.g., in photo-oxidative stress, but are also known to play an important role in eliciting a wide range of defense mechanisms (Baker and Orlandi, 1995; D’Autréaux and Toledano, 2007; **Figure 4C**). One of the earliest plant cellular responses following successful pathogen recognition is the production of ROS, also called oxidative burst (Torres et al., 2006). ROS also play a central role in redox-dependent defense signaling and in creating toxic environments that induce cell death (Dickman and Fluhr, 2013). In this regard, at the cellular juncture of pathogenesis, NPR1, the master regulator of SA-mediated defense, is actually reduced by thioredoxin, which removes the effect of nitrosylation (Tada et al., 2008). Once reduced, the NPR1 oligomer is disrupted and its monomers enter the nucleus, and activate SA-mediated defense and PCD. It should be noted that in a manner that is not fully understood such signaling can take place even under conditions of cellular oxidative stress.

Reactive oxygen species levels can regulate the host cells fate. High ROS levels in plant cells results in a spreading cell death, which provides nutrients to necrotrophic pathogens. Intermediate concentration of ROS usually results in restricted PCD, and low concentration of ROS can act as a signaling molecule, including the activation of antioxidant enzymes usually leading to host protection against necrotrophs (Dickman and Fluhr, 2013). Mitochondria ROS was shown to be involved in plant PCD in both biotic and abiotic stress responses (Dickman and Fluhr, 2013; Tian et al., 2013). A recent report revealed another ROS, singlet oxygen, which plays a major role in plant response to both biotic and abiotic stress (Mor et al., 2014). Signaling of ROS is important in both the ripening process and defense response (**Figure 4C**). However, the exact roles of ROS can appear counterintuitive and there is a need to better understand their compartmentalization.

### Cuticle and Fatty Acid Biosynthesis

Necrotrophic plant pathogens such as *B. cinerea* and *C. gloeosporioides*, at the pathogenic stage, produce cutinases and pectinolytic enzymes to penetrate plant cuticle and epidermal cell wall. Cuticle and cuticular wax are known to regulate fungal cutinase gene expression, leading to the release of cutin monomers from the plant cuticle (Woloshuk and Kolattukudy, 1986). Cutin monomers induce typical PAMP-triggered immunity (PTI) responses including medium alkalization, ethylene production, ROS, and upregulation of defense-related genes (Schweizer et al., 1996; Kauss et al., 1999). Furthermore, treatments with cutin monomers or their production *in vivo* enhances resistance to both biotrophic and necrotrophic fungi (Mengiste et al., 2012). *Arabidopsis* plants expressing a fungal cutinase or mutants with a defective cuticle, such as *long-chain acyl-CoA synthetase2* which is involved in cutin biosynthesis, are surprisingly resistant to *B. cinerea* (Bessire et al., 2007; Chassot et al., 2007). This may be due to faster perception and response to fungal elicitors, easier diffusion of defense signals and antifungal compounds to the infection site and faster oxidative burst (Mengiste et al., 2012). *Bdg* and *lacs2.3* mutants impaired in cutin synthesis are known to display a high level of resistance to *B. cinerea* produced ROS even in the absence of wounding of the leaves (L’Haridon et al., 2011). Moreover, *aba2* and *aba3* mutants together with *bdg* and *lacs2.3* mutants presented increased permeability of the cuticle and enhanced ROS production. In fact, ABA was reported to play an essential role in cuticular permeability, which may influence tomato fruit resistance to *B. cinerea* and may lead to the termination of quiescence (Curvers et al., 2010).

Cuticle related mutants that alter cuticle development and composition were shown to modify plant defenses response and resistance (Voisin et al., 2009). Therefore, the processes of degradation of cuticle and cell wall may have an effect on quiescence. Thus, cell wall and cuticle can constitute valuable targets for improvement of early sensing of pathogen and activation of immune responses accompanied with fruit quality traits. Due to this reason the regulatory mechanisms involved in cuticle deposition have been investigated (Hen-Avivi et al., 2014). The first identified cuticle – associated transcription factor was SHINE1/WAXINDUCER1 (SHN1/WIN; Aharoni et al., 2004; Broun et al., 2004). Recently, it was shown that silencing a tomato ortholog (*SlSHN3*) in the fruit resulted in a dramatic reduction in cuticle formation (Shi et al., 2013). It was suggested in this study that *SlSHN3* regulates not only the genes involved in cutin metabolism but also controls the expression of regulatory genes associated with epidermal cell patterning including tomato genes similar to *GLABRA2* and *MIXTA* (Shi et al., 2013). Recent data showed that *SlMIXTA-like* is a major transcriptional regulator of cutin biosynthesis, likely acting downstream of SlSHINE3. *SlMIXTA-like* not only promoted conical-type epidermal cell development in tomato fruit but also positively regulated cuticular lipids in particular cutin monomer biosynthesis as well as cuticle assembly (Lashbrooke et al., 2015). Tomato fruit silenced in either *SlSHN3* or *SlMIXTA-like* had a significant increase susceptibility to *Colletotrichum* (Shi et al., 2013; Lashbrooke et al., 2015). In another study, leaves of *SlSHN3* over-expressing plants were shown to be more resistant to *B. cinerea* than wild-type leaves, highlighting the importance of cuticle in plant–pathogen interactions (Buxdorf et al., 2014).

The thickness and composition of the cuticle has been shown to influence infection of grape berries by *B. cinerea* (**Figure 2**; Commenil et al., 1997). Hexacosanoic acid, an important component of wax, is present in higher amounts in Touriga Nacional berries than in Trincadeira berries (Agudelo-Romero et al., 2013) and this may be involved in tolerance of Touriga Nacional cultivar to *B. cinerea*. Recently, it was reported that Trincadeira berries infected with *B. cinerea* accumulate saturated long-chain fatty acids which are major constituents of grape waxes accompanied with up-regulation of several acyl-CoA synthetases and wax synthases (Agudelo-Romero et al., 2015). In this study, significant changes were observed in the contents of glycerol and fatty acids such as oleic acid. In addition, a gene encoding a stearyl acyl carrier protein desaturase, which catalyzes the desaturation of stearic acid to oleic acid, was up-regulated in infected berries. It has been shown that a reduction in oleic acid levels results in constitutive activation of the SA-dependent pathway and repression of the JA-dependent pathway (Kachroo and Kachroo, 2009; Kachroo and Robin, 2013). The results in grapes supported these data, since increased oleic acid levels were correlated with activation of JA biosynthesis and signaling in infected berries (Agudelo-Romero et al., 2015). Thus, changes in lipid composition likely represent fruit response to infection.

Interestingly, transcriptome analysis of *C. gloeosporioides* and tomato fruit pathosystem revealed that during appressoria formation and before fungal penetration the tomato fruit host recognizes the fungus and activates fatty acid biosynthesis, elongation, and synthesis of cutin and waxes (Alkan et al., 2015). Specifically, genes involved in synthesis of very long chain fatty acids that are components of cutin and waxes were up-regulated namely 3-ketoacyl CoA synthase and CYP86A cytochrome p450. Interestingly, tomato fruit mutated in CYP86 were much more susceptive to *Colletotrichum* infection (Shi et al., 2013). These genes were also up-regulated in grape berries infected with *B. cinerea* along with a decrease in expression of genes involved in glycerolipid catabolism (Agudelo-Romero et al., 2015). Both pathosystems also reported the induction of β-oxidation fatty acid degradation pathway (Agudelo-Romero et al., 2015; Alkan et al., 2015). This would provide both reducing power and carbon components for metabolism of very long chain fatty acids or alternatively would provide more sugars that might be metabolized by the fungus or serve as precursors of plant secondary metabolites involved in defense. These observations further emphasize the critical role of fatty acids, wax and cutin during infection. This topic deserves further attention, since pathogen and host lipids and lipid metabolites have a critical role in the dynamics of pathogenesis and in plants defense.

### Cell Wall Remodeling and Soluble Sugar Accumulation

Cell walls are structurally complex network of polysaccharides, including cellulose, hemicelluloses, and pectin (Cosgrove, 2005). They serve as a physical barrier that limits pathogen access, but are also involved in pathogen recognition and in the deployment of plant responses to pathogens (Vorwerk et al., 2004; Cantu et al., 2008a,b; Hématy et al., 2009). In order to break down the cell wall barrier pathogens use mechanical force as appressoria (Deising et al., 2000) or release cell wall degrading enzymes (CWDEs), which serve as a pathogenicity factors (Walton, 1994). Also post-harvest pathogens as *Botrytis* (vanKan et al., 1997; Blanco-Ulate et al., 2014) and *Colletotrichum* (Alkan et al., 2013b, 2015) produces CWDEs during pathogenic colonization of tomato fruit. The degradation of the plant cell wall matrix by pathogens may affect the proteins embedded in the cell wall and are likely to activate PAMP-triggered immunity (van Loon et al., 2006; Mengiste, 2012), which often leads to callose deposition at sites of penetration, accumulate phenolic compounds and various toxins in the cell wall and synthesize lignin-like polymers to reinforce the wall (Huckelhoven, 2007).

During natural fleshy fruit ripening the fruit soften as a result of fruit activation of CWDEs as polygalacturonase (PG), pectin methylesterase, pectate lyase, β-galactosidase, cellulase, and expansin (Brummell et al., 1999; Paniagua et al., 2014). Phytohormones as ethylene, ABA, SA and JA, are known to influence the expression of CWDEs which contribute to fruit softening (Huckelhoven, 2007). Because, fruit CWDEs are normally activated during ripening, it was commonly assumed that fruit softening contributes to the transition to susceptibility to pathogens (**Figure 2**; Paniagua et al., 2014). In tomato, the suppression of softening-associated CWDEs, *SlPG* and *SlExp1*, reduced susceptibility to *B. cinerea* infection during ripening, indicating that PG and Exp support both softening (Brummell et al., 1999, 2002; Kalamaki et al., 2003; Powell et al., 2003) and susceptibility to *B. cinerea* (Cantu et al., 2008a,b). Interestingly, *B. cinerea* infections induce *SlPG* and *SlExp1* expression (GonzalezBosch et al., 1996; Flors et al., 2007), suggesting that *Botrytis* induces similar softening to the softening that occurs during fruit ripening. Endo-β-1,4-glucanase (EGase) is another CWDE that have a role in fruit softening. Tomato fruit EGase antisense had enhanced callose deposition and was more resistant against *Botrytis* infection (Flors et al., 2007). Plants PG inhibiting proteins (PGIPs) reduce the pathogen pectin degradation (De Lorenzo et al., 2001). PGIPs inhibit most of the *Botrytis* PGs during pear pathogenesis (Sharrock and Labavitch, 1994). An over-expression of PGIPs enhances ripe tomato fruit tolerance to *Botrytis* (Powell et al., 2000). To conclude, fruit cell wall is a complex and dynamic barrier which changes during ripening and its interaction with fungal pathogens plays a major role in the defense response against pathogens.

### pH Change during Fruit Ripening and Fungal Colonization

The pH change plays an important role in three different aspects of fruit-fungal interaction: (1) the pH change during fruit ripening, (2) fungal-dependent pH modulation of the local infection court, and (3) the local host pH modulation during the activation of defense responses (**Figure 4D**). The combined pH changes were suggested to trigger defense related processes as ROS and activate cell wall hydrolases in the fruit.

The ratio between sugar and pH are determinants of the fruit taste. During fruit ripening total soluble sugars (TSSs) increase and organic acid usually decreases leading to increase in pH. For instance, the pH of avocado fruit increases from 5.2 to 6.0 during ripening (Yakoby et al., 2000). However, in tomato fruit the apoplastic pH decreases during ripening from 6.7 to 4.4 (Almeida and Huber, 1999).

Also fungal pathogens alter their local pH by secretion of ammonia or organic acids to optimize the environment to each fungus enzymatic arsenal (reviewed in Alkan et al., 2013a). Interestingly, fruit pH greatly affects fungal pathogenicity. Acidified environment induce ammonia secretion in alkalizing fungi as *Colletotrichum* and *Alternaria* (Eshel et al., 2002; Alkan et al., 2008), while alkaline environment activate organic acid secretion in acidifying fungi as *Penicillium* and *Botrytis* (Manteau et al., 2003; Hadas et al., 2007). The pH dependent fungal pathogenicity factors are controlled in filamentous fungi by Pal signaling pathway which activates PACC transcription factor (Penalva et al., 2008). PACC activates transcription of those pathogenicity factors at alkaline pH and the AREB transcription factor, which represses acidic expressed pathogenicity factors at alkaline environment (Alkan et al., 2013b; Ment et al., 2015). In this way, fungi adjust their ambient pH in order to optimize the activity of their enzymatic arsenal.

Changes in apoplastic pH, could lead to oxidative burst (Gao et al., 2004). For example, exposing bean cells to alkaline condition resulted in oxidative burst (Wojtaszek et al., 1995). Medium alkalization activate NADPH oxidase, probably as a result of induced K^+^/H^+^ exchange, followed by Ca^2+^ influx/Cl_2_ eﬄux (Simon-Plas et al., 1997; Nurnberger and Scheel, 2001; Zhao et al., 2005). Transient extracellular alkalization is an essential factor in induction of defense response and PCD (Schaller and Oecking, 1999; Clarke et al., 2005; Hano et al., 2008). In this connection, changes in *Arabidopsis thaliana* roots external pH rapidly alter plant gene expression and modulate host responses, similarly to elicitors (Lager et al., 2010). Similarly, tomato fruit apoplastic alkalization by *Colletotrichum* or application of ammonia lead to activation of fruit NADPH oxidase, oxidative burst and SA mediated defense response that ended with extended cell death (Alkan et al., 2012). *P. expansum* secrets gluconic acid and acidify its ambient pH in apples; this acidification was correlated with oxidative burst (Hadas et al., 2007). Taken together both pathogen and fruit modulate their ambient pH in order to optimize respectively their attack and responses (**Figure 4D**).

### Preformed and Inducible Antifungal Resistance

Plants contain preformed secondary metabolites of a defensive nature such as phenolics, sulfur compounds, saponins, cyanogenic glycosides, and glucosinolates. Phenolic compounds play an important role in non-host resistance to fungi. They can either be performed occurring constitutively in healthy plants (phytoanticipins) or synthesized from precursors in response to pathogen attack, being more restricted to the damaged tissue (phytoalexins). Some antibiotic phenolics are stored in plant cells as inactive bound forms but are readily converted into biologically active antibiotics by plant glycosidases in response to pathogen attack. Since these compounds do not involve *de novo* transcription of gene products they are also considered phytoanticipins (Lattanzio et al., 2006). Concentrations of preformed phytoanticipins and inducible phytoalexins were found to decline during fruit ripening (**Figure 2**) and this occurred more rapidly in susceptible cultivars than in resistant cultivars (Prusky, 1996; Lattanzio et al., 2001; Prusky et al., 2013).

The grape berry cultivar Trincadeira is susceptible to *B. cinerea* and downy mildew. It presents lower phenolics content than the tolerant cultivars Touriga Nacional and Aragonês (Ali et al., 2011). The green and *veraison* stages of Aragonês and Touriga Nacional showed higher levels of quercetin glucoside, catechin and hydroxycinnamic acid derivatives such as caftaric acid and coutaric acid than the ripe grape berry (Ali et al., 2011; Agudelo-Romero et al., 2013). A decrease in caffeic acid was also detected in ripe berries of all the three varieties (Agudelo-Romero et al., 2013). This decline may be related to increased susceptibility of ripe fruits to pathogenic fungi. In fact, caffeic acid presents antimicrobial activity (Widmer and Laurent, 2006). Further, constitutive secondary metabolites of the bitter orange *Citrus aurantium* are the flavanone-naringin and the polymethoxyflavone-tangeretin, which showed antifungal activity against *Penicillium digitatum* (Arcas et al., 2000).

Other widely reported preformed antifungal compounds are the family of mono-, di-, and triene compounds in avocado; the resorcinol derivates in mango; the tannins in banana peel (Prusky, 1996) and tomatine in tomato fruits (Itkin et al., 2011). These compounds were shown to decline dramatically during fruit ripening, thus enabling development of penetrating mycelia (Verhoeff, 1974; Prusky et al., 2013).

### Inducible Phenylpropanoid Metabolism

Phytoalexins are generally induced after infection. They accumulate rapidly in response to infection and reach high antimicrobial levels in resistant plants, while there is either lesser or slower accumulation in susceptible plants (Lattanzio et al., 2006). When the accumulation of phytoalexins is either increased or decreased by manipulation of the experimental conditions such as post-harvest stress treatments, the plant and fruit become either more resistant or more susceptible (Lattanzio et al., 2006). Fawe et al. (1998) reported that silicon is involved in the increased resistance of cucumber to powdery mildew by enhancing the antifungal activity due to the presence of metabolites such as flavonol aglycone rhamnetin (3,5,3′,4′-tetrahydroxy-7-*O*-methoxyflavone).

Pathogens often remain quiescent in unripe fruits. During ripening the concentrations of pathogen-induced and pre-formed antifungal phenolics decrease to subtoxic levels (**Figure 2**); this chemical decline occurs more rapidly in susceptible cultivars than in resistant ones (Lattanzio et al., 2006). The principal phenolics in the peach fruit include chlorogenic acid, catechin, and epicatechin. The decline in chlorogenic acid and other endogenous phenolics during fruit ripening correspond to the transition to susceptibility (Bostock et al., 1999). In immature strawberry fruits with a high content of proanthocyanidins *B. cinerea* remains quiescent. When the inhibitory activity of proanthocyanidins in fruits decreases due to maturation, the quiescent fungus can switch to the necrotrophic stage and progress further into the fruit tissue (Jersch et al., 1989). In addition, inducible antifungal compounds, such as capsicannol in pepper, scoparone in citrus, resveratrol in grapes, and others, have been reported to be activated in unripe fruits but not always in ripe fruits (Prusky, 1996). These compounds were hypothesized to be quiescence modulating factors.

The resistance of *Vitis* sp. to *B. cinerea* infection has been shown to correlate with *trans*-resveratrol content (Gabler et al., 2003). Touriga Nacional is not infected by *B. cinerea* under normal field conditions; this cultivar presents higher content in *trans*-resveratrol than the susceptible cultivar Trincadeira (Agudelo-Romero et al., 2013). Recently, in-field infections of Trincadeira cultivar with *B. cinerea* led to profound alterations in secondary metabolism linked to stress response together with a significant increase in *trans*-resveratrol. Indeed, several genes involved in phytoalexin biosynthesis and coding for stilbene synthase and resveratrol synthase were up-regulated at pre-*veraison* in infected grapes (Agudelo-Romero et al., 2015). Therefore, resveratrol was considered a potential positive metabolic marker of *B. cinerea* infection at this stage. Other identified positive markers of infection were gallic acid and 3,4-dihydroxybenzoic acid which present antifungal properties (Lattanzio et al., 2006). In fact, plant benzoic acids and their derivatives are common and widespread mediators of plant responses to biotic and abiotic stress (Wildermuth, 2006). In another work with grape, flavonoid compounds were only found in botrytized berries of botrytized bunches at harvest stage (Hong et al., 2012).

Trincadeira, a *Botrytis*-susceptible variety, is able to initiate to some extent a basal defense reprogramming of the transcriptome and metabolome that is unable to slow down disease progression (Agudelo-Romero et al., 2015). This can be due to the fact that the pathogen can shut down host defenses. For example, sesquiterpenoid biosynthesis, as measured by genes involved in their synthesis; namely *beta*-amyrin synthase and (-)-germacrene D synthase, was down-regulated at the *veraison* stage. Oleanolic acid, a triterpenoid, decreased in infected grapes at pre-*veraison* and *veraison* stages. Unconjugated triterpenoids, such as oleanolic acid, are often found in the epicuticular waxes of plants serving as a first defense barrier against pathogens (Heinzen et al., 1996). This result suggested that infection renders the fruit to be more susceptible by down-regulating defense compounds.

Recently, it was showed through a combined analysis of the transcriptomes of *C. gloeosporioides* and tomato fruit pathosystem that during the quiescent stage, defense pathways were up-regulated including the phenypropanoid pathway for phytoalexin and lignin precursors such as cinnamic, cumarayl, coniferyl, caffeoyl, shikimic, quinic, and sinapyl derivatives (Alkan et al., 2015). The authors suggested that phytoalexin biosynthesis and lignification comprise a major ongoing fruit defense pathway employed by the fruit in response to the persistent presence of quiescent fungi. Such host responses may effectively restrain the pathogen. Indeed, the number of infection sites emerging from quiescence appears to be below the potential primary infection sites, indicating successful containment of the infection. In this study, genes involved in the synthesis of sesquiterpenoids (e.g., rishitin) were also down-regulated together with the up-regulation of key steroid glycoalkaloid (e.g., tomatine) transcripts. Previously, α-tomatine was shown to inhibit *Colletotrichum* fungal growth and germination (Itkin et al., 2011). Hence, transcript expression suggests the occurrence of shifts from rishitin to α -tomatine biosynthetic pathway as an effective response to this fungus. All of those antifungal compounds decline in ripe fruits, which may permit emergence of fungi from quiescence (Prusky, 1996).

## Conclusion

During ripening, fruit undergo major changes such as activation of ethylene synthesis and other phytohormones, pH change, cuticular changes, cell-wall loosening and increase of soluble sugars, decline of antifungal compounds (**Figure 2**), which release the fungus from its quiescent state and promote a necrotrophic and pathogenic life style. Knowledge on the molecular and metabolic events responsible for the onset of necrotrophic stage, occurring both in the host and in fungi, is important key in order to develop strategies to enhance fruit defense and decrease of fungal virulence that ultimately will result in increased quality of fruits. This knowledge can be considered in breeding programs, pre and post-harvest treatments or alternatively provide a framework for biotechnological approaches.

## Conflict of Interest Statement

The authors declare that the research was conducted in the absence of any commercial or financial relationships that could be construed as a potential conflict of interest.

## References

[B1] AbuQamar S. Chen X. Dhawan R. Bluhm B. Salmeron J. Lam S. (2006). Expression profiling and mutant analysis reveals complex regulatory networks involved in *Arabidopsis* response to Botrytis infection. *Plant J.* 48 28–44. 10.1111/j.1365-313X.2006.02849.x 16925600

[B2] Adaskaveg J. E. Forster H. Thompson D. F. (2000). Identification and etiology of visible quiescent infections of *Monilinia fructicola* and *Botrytis cinerea* in sweet cherry fruit. *Plant Dis.* 84 328–333. 10.1094/PDIS.2000.84.3.328 30841251

[B3] Agudelo-Romero P. Erban A. Rego C. Carbonell-Bejerano P. Nascimento T. Sousa L. (2015). Transcriptome and metabolome reprogramming in *Vitis vinifera* cv. *Trincadeira berries* upon infection with *Botrytis cinerea*. *J. Exp. Bot.* 66 1769–1785. 10.1093/jxb/eru517 25675955 PMC4669548

[B4] Agudelo-Romero P. Erban A. Sousa L. Pais M. S. Kopka J. Fortes A. M. (2013). Search for transcriptional and metabolic markers of grape pre-ripening and ripening and insights into specific aroma development in three portuguese cultivars. *PLoS ONE* 8:e60422 10.1371/journal.pone.0060422 PMC3614522 23565246

[B5] Aharoni A. Dixit S. Jetter R. Thoenes E. van Arkel G. Pereira A. (2004). The SHINE clade of AP2 domain transcription factors activates wax biosynthesis, alters cuticle properties, and confers drought tolerance when overexpressed in *Arabidopsis*. *Plant Cell* 16 2463–2480. 10.1105/tpc.104.022897 15319479 PMC520946

[B6] Alexander L. Grierson D. (2002). Ethylene biosynthesis and action in tomato: a model for climacteric fruit ripening. *J. Exp. Bot.* 53 2039–2055. 10.1093/jxb/erf072 12324528

[B7] Ali K. Maltese F. Fortes A. M. Pais M. S. Choi Y. H. Verpoorte R. (2011). Monitoring biochemical changes during grape berry development in *Portuguese cultivars* by NMR spectroscopy. *Food Chem.* 124 1760–1769. 10.1016/j.foodchem.2010.08.015

[B8] Alkan N. Davydov O. Sagi M. Fluhr R. Prusky D. (2009). Ammonium secretion by *Colletotrichum coccodes* activates host NADPH oxidase activity enhancing host cell death and fungal virulence in tomato fruits. *Mol. Plant Microbe* 22 1484–1491. 10.1094/MPMI-22-12-1484 19888814

[B9] Alkan N. Espeso E. A. Prusky D. (2013a). Virulence regulation of phytopathogenic fungi by pH. *Antioxid. Redox Signal.* 19 1012–1025. 10.1089/ars.2012.5062 23249178

[B10] Alkan N. Meng X. Friedlander G. Reuveni E. Sukno S. Sherman A. (2013b). Global aspects of pacC regulation of pathogenicity genes in *Colletotrichum gloeosporioides* as revealed by transcriptome analysis. *Mol. Plant Microbe* 26 1345–1358. 10.1094/MPMI-03-13-0080-R 23902260

[B11] Alkan N. Fluhr R. Prusky D. (2012). Ammonium secretion during *Colletotrichum coccodes* infection modulates salicylic and jasmonic acid pathways of ripe and unripe tomato fruit. *Mol. Plant Microbe* 25 85–96. 10.1094/MPMI-01-11-0020 22150075

[B12] Alkan N. Fluhr R. Sherman A. Prusky D. (2008). Role of ammonia secretion and pH modulation on pathogenicity of *Colletotrichum coccodes* on tomato fruit. *Mol. Plant Microbe* 21 1058–1066. 10.1094/MPMI-21-8-1058 18616402

[B13] Alkan N. Friedlander G. Ment D. Prusky D. Fluhr R. (2015). Simultaneous transcriptome analysis of *Colletotrichum gloeosporioides* and tomato fruit pathosystem reveals novel fungal pathogenicity and fruit defense strategies. *New Phytol.* 205 801–815. 10.1111/nph.13087 25377514

[B14] Almeida D. P. Huber D. J. (1999). Apoplastic pH and inorganic ion levels in tomato fruit: a potential means for regulation of cell wall metabolism during ripening. *Physiol. Plant.* 105 506–512. 10.1034/j.1399-3054.1999.105316.x

[B15] Arcas M. C. Botia J. M. Ortuno A. M. Del Rio J. A. (2000). UV irradiation alters the levels of flavonoids involved in the defence mechanism of *Citrus aurantium* fruits against *Penicillium digitatum*. *Eur. J. Plant Pathol.* 106 617–622. 10.1023/A:1008704102446

[B16] Aver’Yanov A. A. Belozerskaya T. A. Gessler N. N. (2012). *Fungus Development and Reactive Oxygen: Phytopathological Aspects, Biocommunication of Fungi* (Berlin: Springer) 261–271.

[B17] Baker C. J. Orlandi E. W. (1995). Active oxygen in plant pathogenesis. *Annu. Rev. Phytopathol.* 33 299–321. 10.1146/annurev.py.33.090195.001503 18999963

[B18] Bargel H. Neinhuis C. (2005). Tomato (*Lycopersicon esculentum* Mill.) fruit growth and ripening as related to the biomechanical properties of fruit skin and isolated cuticle. *J. Exp. Bot.* 56 1049–1060. 10.1093/jxb/eri098 15710631

[B19] Bari R. Jones J. (2009). Role of plant hormones in plant defence responses. *Plant Mol. Biol.* 69 473–488. 10.1007/s11103-008-9435-0 19083153

[B20] Barkai-Golan R. (2001). *Postharvest Diseases of Fruits and Vegetables: Development and Control.* Amsterdam: Elsevier.

[B21] Beno-Moualem D. Prusky D. (2000). Early events during quiescent infection development by *Colletotrichum gloeosporioides* in unripe avocado fruits. *Phytopathology* 90 553–559. 10.1094/PHYTO.2000.90.5.553 18944563

[B22] Bessire M. Chassot C. Jacquat A. C. Humphry M. Borel S. Petétot J. M. C. (2007). A permeable cuticle in *Arabidopsis* leads to a strong resistance to *Botrytis cinerea*. *EMBO J.* 26 2158–2168. 10.1038/sj.emboj.7601658 17396154 PMC1852784

[B23] Biswas P. East A. Hewett E. Heyes J. (2014). Ripening delay caused by 1-MCP may increase tomato chilling sensitivity. *N. Z. J. Crop Hortic. Sci.* 42 145–150. 10.1080/01140671.2013.870218

[B24] Biton E. Kobiler I. Feygenberg O. Yaari M. Friedman H. Prusky D. (2014). Control of alternaria black spot in persimmon fruit by a mixture of gibberellin and benzyl adenine, and its mode of action. *Postharvest Biol. Technol.* 94 82–88. 10.1016/j.postharvbio.2014.03.009

[B25] Bitrian M. Zarza X. Altabella T. Tiburcio A. F. Alcazar R. (2012). Polyamines under abiotic stress: metabolic crossroads and hormonal crosstalks in plants. *Metabolites* 2 516–528. 10.3390/metabo2030516 24957645 PMC3901213

[B26] Blanco-Ulate B. Morales-Cruz A. Amrine K. Labavitch J. M. Powell A. Cantu D. (2014). Genome-wide transcriptional profiling of *Botrytis cinerea* genes targeting plant cell walls during infections of different hosts. *Front. Plant Sci.* 5:435 10.3389/fpls.2014.00435 PMC4153048 25232357

[B27] Blanco-Ulate B. Vincenti E. Powell A. L. T. Cantu D. (2013). Tomato transcriptome and mutant analyses suggest a role for plant stress hormones in the interaction between fruit and *Botrytis cinerea*. *Front. Plant Sci.* 4:142 10.3389/fpls.2013.00142 PMC3653111 23717322

[B28] Bostock R. M. Wilcox S. M. Wang G. Adaskaveg J. E. (1999). Suppression of *Monilinia fructicola* cutinase production by peach fruit surface phenolic acids. *Physiol. Mol. Plant Pathol.* 54 37–50. 10.1006/pmpp.1998.0189

[B29] Brauc S. De Vooght E. Claeys M. Geuns J. M. C. Hofte M. Angenon G. (2012). Overexpression of arginase in *Arabidopsis thaliana* influences defence responses against *Botrytis cinerea*. *Plant Biol.* 14 39–45. 10.1111/j.1438-8677.2011.00520.x 22188168

[B30] Brennan T. Frenkel C. (1977). Involvement of hydrogen peroxide in the regulation of senescence in pear. *Plant Physiol.* 59 411–416. 10.1104/pp.59.3.411 16659863 PMC542414

[B31] Broun P. Poindexter P. Osborne E. Jiang C. Z. Riechmann J. L. (2004). WIN1 a transcriptional activator of epidermal wax accumulation in *Arabidopsis*. *Proc. Natl. Acad. Sci. U.S.A.* 101 4706–4711. 10.1073/pnas.0305574101 15070782 PMC384811

[B32] Brummell D. A. Harpster M. H. Civello P. M. Palys J. M. Bennett A. B. Dunsmuir P. (1999). Modification of expansin protein abundance in tomato fruit alters softening and cell wall polymer metabolism during ripening. *Plant Cell* 11 2203–2216. 10.1105/tpc.11.11.2203 10559444 PMC144123

[B33] Brummell D. A. Howie W. J. Ma C. Dunsmuir P. (2002). Postharvest fruit quality of transgenic tomatoes suppressed in expression of a ripening-related expansin. *Postharvest Biol. Technol.* 25 209–220. 10.1016/S0925-5214(01)00179-X

[B34] Burg S. P. Burg E. A. (1965). Ethylene action and the ripening of fruits ethylene influences the growth and development of plants and is the hormone which initiates fruit ripening. *Science* 148 1190–1196. 10.1126/science.148.3674.1190 14280001

[B35] Buxdorf K. Rubinsky G. Barda O. Burdman S. Aharoni A. Levy M. (2014). The transcription factor SlSHINE3 modulates defense responses in tomato plants. *Plant Mol. Biol.* 84 37–47. 10.1007/s11103-013-0117-1 23943056

[B36] Buzby J. C. Wells H. F. Hyman J. (2014). *The Estimated Amount, Value, and Calories of Postharvest Food Losses at the Retail and Consumer Levels in the United States*. USDA EIB-121, US. Department of Agriculture, Economic Research Service. 10.2139/ssrn.2501659

[B37] Cannon P. F. Bridge P. D. Monte E. (2000). “Linking the past, present, and future of Colletotrichum systematics,” in *‘Colletotrichum: Host Specificity, Pathology, and Host Pathogen Interaction* eds Prusky D. F. S. Dickman M. B. (St. Paul, MI: APS Presspp) 1–20.

[B38] Cantu D. Blanco-Ulate B. Yang L. Labavitch J. M. Bennett A. B. Powell A. L. T. (2009). Ripening-regulated susceptibility of tomato fruit to *Botrytis cinerea* requires NOR but not RIN or ethylene. *Plant Physiol.* 150 1434–1449. 10.1104/pp.109.138701 19465579 PMC2705034

[B39] Cantu D. Vicente A. R. Greve L. C. Dewey F. M. Bennett A. B. Labavitch J. M. (2008a). The intersection between cell wall disassembly, ripening, and fruit susceptibility to *Botrytis cinerea*. *Proc. Natl. Acad. Sci. U.S.A.* 105 859–864. 10.1073/pnas.0709813105 18199833 PMC2242701

[B40] Cantu D. Vicente A. R. Labavitch J. M. Bennett A. B. Powell A. L. T. (2008b). Strangers in the matrix: plant cell walls and pathogen susceptibility. *Trends Plant Sci.* 13 610–617. 10.1016/j.tplants.2008.09.002 18824396

[B41] Cessna S. G. Sears V. E. Dickman M. B. Low P. S. (2000). Oxalic acid, a pathogenicity factor for *Sclerotinia sclerotiorum*, suppresses the oxidative burst of the host plant. *Plant Cell* 12 2191–2199. 10.1105/tpc.12.11.2191 11090218 PMC150167

[B42] Chassot C. Nawrath C. Metraux J. P. (2007). Cuticular defects lead to full immunity to a major plant pathogen. *Plant J.* 49 972–980. 10.1111/j.1365-313X.2006.03017.x 17257167

[B43] Clarke A. Mur L. A. J. Darby R. M. Kenton P. (2005). Harpin modulates the accumulation of salicylic acid by *Arabidopsis* cells via apoplastic alkalization. *J. Exp. Bot.* 56 3129–3136. 10.1093/jxb/eri310 16246855

[B44] Commenil P. Brunet L. Audran J.-C. (1997). The development of the grape berry cuticle in relation to susceptibility to bunch rot disease. *J. Exp. Bot.* 48 1599–1607. 10.1093/jexbot/48.313.1599

[B45] Cosgrove D. J. (2005). Growth of the plant cell wall. *Nat. Rev. Mol. Cell Biol.* 6 850–861. 10.1038/nrm1746 16261190

[B46] Curvers K. Seifi H. Mouille G. de Rycke R. Asselbergh B. Van Hecke A. (2010). Abscisic acid deficiency causes changes in cuticle permeability and pectin composition that influence tomato resistance to *Botrytis cinerea*. *Plant Physiol.* 154 847–860. 10.1104/pp.110.158972 20709830 PMC2949027

[B47] D’Autréaux B. Toledano M. B. (2007). ROS as signalling molecules: mechanisms that generate specificity in ROS homeostasis. *Nat. Rev. Mol. Cell Biol.* 8 813–824. 10.1038/nrm2256 17848967

[B48] De Lorenzo G. D’Ovidio R. Cervone F. (2001). The role of polygalacturonase-inhibiting proteins (PGIPS) in defense against pathogenic fungi. *Annu. Rev. Phytopathol.* 39 313–335. 10.1146/annurev.phyto.39.1.313 11701868

[B49] Deising H. B. Werner S. Wernitz M. (2000). The role of fungal appressoria in plant infection. *Microbes Infect.* 2 1631–1641. 10.1016/S1286-4579(00)01319-8 11113382

[B50] Dickman M. B. Fluhr R. (2013). Centrality of host cell death in plant-microbe interactions. *Annu. Rev. Phytopathol.* 51 543–570. 10.1146/annurev-phyto-081211-173027 23915134

[B51] Dou H. Jones S. Ritenour M. (2005). Influence of 1-MCP application and concentration on post-harvest peel disorders and incidence of decay in citrus fruit. *J. Hortic. Sci. Biotechnol.* 80 786–792.

[B52] Emery K. M. Michailides T. J. Scherm H. (2000). Incidence of latent infection of immature peach fruit by *Monilinia fructicola* and relationship to brown rot in Georgia. *Plant Dis.* 84 853–857. 10.1094/PDIS.2000.84.8.853 30832138

[B53] Eshel D. Ben-Arie R. Dinoor A. Prusky D. (2000). Resistance of gibberellin-treated persimmon fruit to *Alternaria alternata* arises from the reduced ability of the fungus to produce endo-1,4-beta-glucanase. *Phytopathology* 90 1256–1262. 10.1094/PHYTO.2000.90.11.1256 18944429

[B54] Eshel D. Miyara I. Ailing T. Dinoor A. Prusky D. (2002). pH regulates endoglucanase expression and virulence of *Alternaria alternata* persimmon fruit. *Mol. Plant Microbe* 15 774–779. 10.1094/MPMI.2002.15.8.774 12182334

[B55] Fawe A. Abou-Zaid M. Menzies J. G. Belanger R. R. (1998). Silicon-mediated accumulation of flavonoid phytoalexins in cucumber. *Phytopathology* 88 396–401. 10.1094/PHYTO.1998.88.5.396 18944917

[B56] Flors V. Leyva M. D. Vicedo B. Finiti I. Real M. D. Garcia-Agustin P. (2007). Absence of the endo-beta-1,4-glucanases Cel1 and Cel2 reduces susceptibility to *Botrytis cinerea* in tomato. *Plant J.* 52 1027–1040. 10.1111/j.1365-313X.2007.03299.x 17916112

[B57] Fujita M. Fujita Y. Noutoshi Y. Takahashi F. Narusaka Y. Yamaguchi-Shinozaki K. (2006). Crosstalk between abiotic and biotic stress responses: a current view from the points of convergence in the stress signaling networks. *Curr. Opin. Plant Biol.* 9 436–442. 10.1016/j.pbi.2006.05.014 16759898

[B58] Gabler F. M. Smilanick J. L. Mansour M. Ramming D. W. Mackey B. E. (2003). Correlations of morphological, anatomical, and chemical features of grape berries with resistance to *Botrytis cinerea*. *Phytopathology* 93 1263–1273. 10.1094/PHYTO.2003.93.10.1263 18944326

[B59] Gao D. Knight M. R. Trewavas A. J. Sattelmacher B. Plieth C. (2004). Self-reporting *Arabidopsis* expressing pH and [Ca^2+^] indicators unveil ion dynamics in the cytoplasm and in the apoplast under abiotic stress. *Plant Physiol.* 134 898–908. 10.1104/pp.103.032508 15020753 PMC389913

[B60] Geny L. Darrieumerlou A. Doneche B. (2003). Conjugated polyamines and hydroxycinnamic acids in grape berries during *Botrytis cinerea* disease development: differences between ‘noble rot’ and ‘grey mould’. *Austr. J. Grape Wine Res.* 9 102–106. 10.1111/j.1755-0238.2003.tb00259.x

[B61] Giovannoni J. (2001). Molecular biology of fruit maturation and ripening. *Annu. Rev. Plant Phys.* 52 725–749. 10.1146/annurev.arplant.52.1.725 11337414

[B62] Glazebrook J. (2005). Contrasting mechanisms of defense against biotrophic and necrotrophic pathogens. *Annu. Rev. Phytopathol.* 43 205–227. 10.1146/annurev.phyto.43.040204.135923 16078883

[B63] GonzalezBosch C. Brummell D. A. Bennett A. B. (1996). Differential expression of two endo-1,4-beta-glucanase genes in pericarp and locules of wild-type and mutant tomato fruit. *Plant Physiol.* 111 1313–1319. 12226364 10.1104/pp.111.4.1313 PMC161016

[B64] Griebel T. Zeier J. (2010). A role for beta-sitosterol to stigmasterol conversion in plant-pathogen interactions. *Plant J.* 63 254–268. 10.1111/j.1365-313X.2010.04235.x 20444228

[B65] Gustavsson J. Cederberg C. Sonesson U. van Otterdijk R. Meybeck A. (2011). *Global Food Losses and Food Waste - Extent, Causes and Prevention.* Rome: Food and Agriculture Organization.

[B66] Hadas Y. Goldberg I. Pines O. Prusky D. (2007). Involvement of gluconic acid and glucose oxidase in the pathogenicity of *Penicillium expansum* in apples. *Phytopathology* 97 384–390. 10.1094/PHYTO-97-3-0384 18943660

[B67] Hano C. Addi M. Fliniaux O. Bensaddek L. Duverger E. Mesnard F. (2008). Molecular characterization of cell death induced by a compatible interaction between *Fusarium oxysporum* f. sp linii and flax *(Linum usitatissimum) cells*. *Plant Physiol. Biochem.* 46 590–600. 10.1016/j.plaphy.2008.02.004 18396055

[B68] Harberd N. P. Belfield E. Yasumura Y. (2009). The angiosperm gibberellin-GID1-DELLA growth regulatory mechanism: how an “Inhibitor of an Inhibitor” enables flexible response to fluctuating environments. *Plant Cell* 21 1328–1339. 10.1105/tpc.109.066969 19470587 PMC2700538

[B69] Heinzen H. deVries J. X. Moyna P. Remberg G. Martinez R. Tietze L. F. (1996). Mass spectrometry of labelled triterpenoids: thermospray and electron impact ionization analysis. *Phytochem. Anal.* 7 237–244. 10.1002/(SICI)1099-1565(199609)7:5<237::AID-PCA310>3.0.CO;2-M

[B70] Hématy K. Cherk C. Somerville S. (2009). Host–pathogen warfare at the plant cell wall. *Curr. Opin. Plant Biol.* 12 406–413. 10.1016/j.pbi.2009.06.007 19616468

[B71] Hen-Avivi S. Lashbrooke J. Costa F. Aharoni A. (2014). Scratching the surface: genetic regulation of cuticle assembly in fleshy fruit. *J. Exp. Bot.* 65 4653–4664. 10.1093/jxb/eru225 24916070

[B72] Hodges D. (2003). *Overview: Oxidative Stress and Postharvest Produce. Postharvesst Oxidative Stress in Horticultural Crops* (New York, NY: Food Products Press) 1–12.

[B73] Hofman P. J. Jobin-Decor M. Meiburg G. F. Macnish A. J. Joyce D. C. (2001). Ripening and quality responses of avocado, custard apple, mango and papaya fruit to 1-methylcyclopropene. *Aust. J. Exp. Agric.* 41 567–572. 10.1071/EA00152

[B74] Hong Y. Martinez A. Liger-Belair G. Jeandet P. Nuzillard J. Cilindre C. (2012). Metabolomics reveals simultaneous influences of plant defence system and fungal growth in *Botrytis cinerea*-infected *Vitis vinifera* cv. *Chardonnay berries. J. Exp. Bot.* 63 5773–5785. 10.1093/jxb/ers228 22945941

[B75] Huckelhoven R. (2007). Cell wall - associated mechanisms of disease resistance and susceptibility. *Annu. Rev. Phytopathol.* 45 101–127. 10.1146/annurev.phyto.45.062806.094325 17352660

[B76] Hyde K. D. Cai L. Cannon P. F. Crouch J. A. Crous P. W. Damm U. (2009). Colletotrichum - names in current use. *Fungal Divers* 39 147–182.

[B77] Itkin M. Rogachev I. Alkan N. Rosenberg T. Malitsky S. Masini L. (2011). GLYCOALKALOID METABOLISM1 is required for steroidal alkaloid glycosylation and prevention of phytotoxicity in tomato. *Plant Cell* 23 4507–4525. 10.1105/tpc.111.088732 22180624 PMC3269880

[B78] Janisiewicz W. J. Leverentz B. Conway W. S. Saftner R. A. Reed A. N. Camp M. J. (2003). Control of bitter rot and blue mold of apples by integrating heat and antagonist treatments on 1-MCP treated fruit stored under controlled atmosphere conditions. *Postharvest Biol. Technol.* 29 129–143. 10.1016/S0925-5214(03)00040-1

[B79] Jersch S. Scherer C. Huth G. Schlosser E. (1989). Proanthocyanidins as basis for quiescence of *Botrytis-cinerea* in immature strawberry fruits. *Z. Pflanzenk. Pflanzen* 96 365–378.

[B80] Johnson G. I. Mead A. J. Cooke A. W. Dean J. R. (1992). Mango stem end rot pathogens - fruit infection by endophytic colonization of the inflorescence and pedicel. *Ann. Appl. Biol.* 120 225–234. 10.1111/j.1744-7348.1992.tb03420.x

[B81] Kachroo A. Kachroo P. (2009). Fatty acid-derived signals in plant defense. *Annu. Rev. Phytopathol.* 47 153–176. 10.1146/annurev-phyto-080508-081820 19400642

[B82] Kachroo A. Robin G. P. (2013). Systemic signaling during plant defense. *Curr. Opin. Plant Biol.* 16 527–533. 10.1016/j.pbi.2013.06.019 23870750

[B83] Kalamaki M. S. Harpster M. H. Palys J. M. Labavitch J. M. Reid D. S. Brummell D. A. (2003). Simultaneous transgenic suppression of LePG and LeExp1 influences rheological properties of juice and concentrates from a processing tomato variety. *J. Agric. Food Chem.* 51 7456–7464. 10.1021/jf034164l 14640599

[B84] Kauss H. Fauth M. Merten A. Jeblick W. (1999). Cucumber hypocotyls respond to cutin monomers via both an inducible and a constitutive H2O2-generating system. *Plant Physiol.* 120 1175–1182. 10.1104/pp.120.4.1175 10444101 PMC59351

[B85] Kemmerling B. Schwedt A. Rodriguez P. Mazzotta S. Frank M. Abu Qamar S. (2007). The BRI1-associated kinase 1, BAK1, has a Brassinoli-independent role in plant cell-death control. *Curr. Biol.* 17 1116–1122. 10.1016/j.cub.2007.05.046 17583510

[B86] Kim K. S. Min J. Y. Dickman M. B. (2008). Oxalic acid is an elicitor of plant programmed cell death during *Sclerotinia sclerotiorum* disease development. *Mol. Plant Microbe* 21 605–612. 10.1094/MPMI-21-5-0605 18393620

[B87] Krishna P. (2003). Brassinosteroid-mediated stress responses. *J. Plant Growth Regul.* 22 289–297. 10.1007/s00344-003-0058-z 14676968

[B88] Ku V. V. V. Wills R. B. H. Ben-Yehoshua S. (1999). 1-methylcyclopropene can differentially affect the postharvest life of strawberries exposed to ethylene. *Hortscience* 34 119–120.

[B89] Lacan D. Baccou J.-C. (1998). High levels of antioxidant enzymes correlate with delayed senescence in nonnetted muskmelon fruits. *Planta* 204 377–382. 10.1007/s004250050269

[B90] Lager I. Andréasson O. Dunbar T. L. Andreasson E. Escobar M. A. Rasmusson A. G. (2010). Changes in external pH rapidly alter plant gene expression and modulate auxin and elicitor responses. *Plant Cell Environ.* 33 1513–1528. 10.1111/j.1365-3040.2010.02161.x 20444216 PMC2920358

[B91] Lashbrooke J. G. Adato A. Alkan N. Tsimbalist T. Rechav K. Fernandez J. P. (2015). The tomato MIXTA-like transcription factor coordinates fruit epidermis conical cell development and cuticular lipid biosynthesis and assembly. *Plant Physiol.* 10.1104/pp.15.01145 [Epub ahead of print]. PMC4677903 26443676

[B92] Lattanzio V. Di Venere D. Linsalata V. Bertolini P. Ippolito A. Salermo M. (2001). Low temperature metabolism of apple phenolics and quiescence of *Phlyctaena vagabunda*. *J. Agric. Food Chem.* 49 5817–5821. 10.1021/jf010255b 11743768

[B93] Lattanzio V. Lattanzio V. M. T. Cardinali A. (2006). “Role of phenolics in the resistance mechanisms of plants against fungal pathogens and insects,” in *Phytochemistry: Advances in Research* ed. Imperato F. (Kerala: Research Signpost) 23–67.

[B94] Lester G. (2003). *Oxidative Stress Affecting Fruit Senescence. Postharvest Oxidative Stress in Horticultural Crops* (New York: Food Products Press) 113–129.

[B95] L’Haridon F. Besson-Bard A. Binda M. Serrano M. Abou-Mansour E. Balet F. (2011). A permeable cuticle is associated with the release of reactive oxygen species and induction of innate immunity. *PLoS Pathog.* 7:e1002148 10.1371/journal.ppat.1002148 PMC3145797 21829351

[B96] Lipinski B. Hanson C. Lomax J. Kitinoja L. Raite W. Searchinger T. (2013). *Reducing Food Loss and Waste.* Washington, DC: United Nations Environment Programme.

[B97] Liu H. X. Jiang W. B. Zhou L. G. Wang B. G. Luo Y. B. (2005). The effects of 1-methylcyclopropene on peach fruit (*Prunus persica* L. cv. Jiubao) ripening and disease resistance. *Int. J. Food Sci. Technol.* 40 1–7. 10.1111/j.1365-2621.2004.00905.x

[B98] Manteau S. Abouna S. Lambert B. Legendre L. (2003). Differential regulation by ambient pH of putative virulence factor secretion by the phytopathogenic fungus *Botrytis cinerea*. *FEMS Microbiol. Ecol.* 43 359–366. 10.1111/j.1574-6941.2003.tb01076.x 19719667

[B99] Marcos J. F. González-Candelas L. Zacarías L. (2005). Involvement of ethylene biosynthesis and perception in the susceptibility of citrus fruits to *Penicillium digitatum* infection and the accumulation of defence-related mRNAs. *J. Exp. Bot.* 56 2183–2193. 10.1093/jxb/eri218 15983011

[B100] Mengiste T. (2012). Plant immunity to necrotrophs. *Annu. Rev. Phytopathol.* 50 267–294. 10.1146/annurev-phyto-081211-172955 22726121

[B101] Mengiste T. VanAlfen N. Leach J. Lindow S. (2012). Plant immunity to necrotrophs. *Annu. Rev. Phytopathol.* 50 267–294. 10.1146/annurev-phyto-081211-172955 22726121

[B102] Menniti A. M. Gregori R. Donati I. (2004). 1-methylcyclopropene retards postharvest softening of plums. *Postharvest Biol. Technol.* 31 269–275. 10.1016/j.postharvbio.2003.09.009

[B103] Ment D. Alkan N. Luria N. Bi F.-C. Reuveni E. Fluhr R. (2015). A role of AREB in the regulation of PACC-dependent acid-expressed-genes and pathogenicity of *Colletotrichum gloeosporioides*. *Mol. Plant Microbe* 28 154–166. 10.1094/MPMI-09-14-0252-R 25317668

[B104] Mor A. Koh E. Weiner L. Rosenwasser S. Sibony-Benyamini H. Fluhr R. (2014). Singlet oxygen signatures are detected independent of light or chloroplasts in response to multiple stresses. *Plant Physiol.* 165 249–261. 10.1104/pp.114.236380 24599491 PMC4012584

[B105] Navarro L. Bari R. Achard P. Lison P. Nemri A. Harberd N. P. (2008). DELLAs control plant immune responses by modulating the balance of jasmonic acid and salicylic acid signaling. *Curr. Biol.* 18 650–655. 10.1016/j.cub.2008.03.060 18450451

[B106] Nurnberger T. Scheel D. (2001). Signal transmission in the plant immune response. *Trends Plant Sci.* 6 372–379. 10.1016/S1360-1385(01)02019-2 11495791

[B107] O’Connell R. J. Thon M. R. Hacquard S. Amyotte S. G. Kleemann J. Torres M. F. (2012). Lifestyle transitions in plant pathogenic *Colletotrichum fungi* deciphered by genome and transcriptome analyses. *Nat. Genet.* 44 1060–1065. 10.1038/ng.2372 22885923 PMC9754331

[B108] Okawa K. (2014). *Market and Trade Impacts of Food Loss and Waste Reduction* (Paris: Organisation for Economic Co-operation and Development).

[B109] Osorio S. Alba R. Damasceno C. M. Lopez-Casado G. Lohse M. Zanor M. I. (2011). Systems biology of tomato fruit development: combined transcript, protein, and metabolite analysis of tomato transcription factor (nor, rin) and ethylene receptor (Nr) mutants reveals novel regulatory interactions. *Plant Physiol.* 157 405–425. 10.1104/pp.111.175463 21795583 PMC3165888

[B110] Paniagua C. Pose S. Morris V. J. Kirby A. R. Quesada M. A. Mercado J. A. (2014). Fruit softening and pectin disassembly: an overview of nanostructural pectin modifications assessed by atomic force microscopy. *Ann. Bot. Lond.* 114 1375–1383. 10.1093/aob/mcu149 PMC4195560 25063934

[B111] Penalva M. A. Tilburn J. Bignell E. Arst H. N. (2008). Ambient pH gene regulation in fungi: making connections. *Trends Microbiol.* 16 291–300. 10.1016/j.tim.2008.03.006 18457952

[B112] Pieterse C. M. J. Van der Does D. Zamioudis C. Leon-Reyes A. Van Wees S. C. M. (2012). Hormonal modulation of plant immunity. *Annu. Rev. Cell Dev. Biol.* 28 489–521. 10.1146/annurev-cellbio-092910-154055 22559264

[B113] Porat R. Weiss B. Cohen L. Daus A. Goren R. Droby S. (1999). Effects of ethylene and 1-methylcyclopropene on the postharvest qualities of ‘Shamouti’ oranges. *Postharvest Biol. Technol.* 15 155–163. 10.1016/S0925-5214(98)00079-9

[B114] Powell A. L. T. Kalamaki M. S. Kurien P. A. Gurrieri S. Bennett A. B. (2003). Simultaneous transgenic suppression of LePG and LeExp1 influences fruit texture and juice viscosity in a fresh market tomato variety. *J. Agric. Food Chem.* 51 7450–7455. 10.1021/jf034165d 14640598

[B115] Powell A. L. T. van Kan J. ten Have A. Visser J. Greve L. C. Bennett A. B. (2000). Transgenic expression of pear PGIP in tomato limits fungal colonization. *Mol. Plant Microbe* 13 942–950. 10.1094/MPMI.2000.13.9.942 10975651

[B116] Prins T. W. Tudzynski P. von Tiedemann A. Tudzynski B. ten Have A. Hansen M. E. (2000). “Infections strategies of *Botrytis cinerea* and related necrotrophic pathogens,” in *Fungal Pathology* ed. Kronstad W. (Dordrecht: Kluwer Academic) 33–65. 10.1007/978-94-015-9546-9_2

[B117] Prusky D. (1996). Pathogen quiescence in postharvest diseases. *Annu. Rev. Phytopathol.* 34 413–434. 10.1146/annurev.phyto.34.1.413 15012550

[B118] Prusky D. Alkan N. Mengiste T. Fluhr R. (2013). Quiescent and necrotrophic lifestyle choice during postharvest disease development. *Annu. Rev. Phytopathol.* 51 55–76. 10.1146/annurev-phyto-082712-102349 23682917

[B119] Prusky D. Benarie R. Guelfatreich S. (1981). Etiology and histology of Alternaria rot of persimmon fruits. *Phytopathology* 71 1124–1128. 10.1094/Phyto-71-1124

[B120] Prusky D. Kobiler I. Miyara I. Alkan N. (2009). “Fruit diseases,” in *The Mango, Botany, Production and Uses* 2nd Edn ed. Litz R. E. (Cambridge: CABI International) 210–231. 10.1079/9781845934897.0210

[B121] Rijkenberg F. Leeuw G. D. Verhoeff K. (1980). Light and electron microscopy studies on the infection of tomato fruits by *Botrytis cinerea*. *Can. J. Bot.* 58 1394–1404. 10.1139/b80-170

[B122] Robert-Seilaniantz A. Grant M. Jones J. D. G. (2011). Hormone crosstalk in plant disease and defense: more than just jasmonate-salicylate antagonism. *Annu. Rev. Phytopathol.* 49 317–343. 10.1146/annurev-phyto-073009-114447 21663438

[B123] Rogiers S. Y. Kumar G. N. M. Knowles N. R. (1998). Maturation and ripening of fruit of *Amelanchier alnifolia* Nutt are accompanied by increasing oxidative stress. *Ann. Bot. Lond.* 81 203–211. 10.1006/anbo.1997.0543

[B124] Saftner R. A. Abbott J. A. Conway W. S. Barden C. L. (2003). Effects of 1-methylcyclopropene and heat treatments on ripening and postharvest decay in ‘Golden Delicious’ apples. *J. Am. Soc. Hortic. Sci.* 128 120–127.

[B125] Schaller A. Oecking C. (1999). Modulation of plasma membrane H+-ATPase activity differentially activates wound and pathogen defense responses in tomato plants. *Plant Cell* 11 263–272. 9927643 10.1105/tpc.11.2.263 PMC144172

[B126] Schweizer P. Felix G. Buchala A. Müller C. Métraux J. P. (1996). Perception of free cutin monomers by plant cells. *Plant J.* 10 331–341. 10.1046/j.1365-313X.1996.10020331.x

[B127] Seymour G. B. Østergaard L. Chapman N. H. Knapp S. Martin C. (2013). Fruit development and ripening. *Annu. Rev. Plant Biol.* 64 219–241. 10.1146/annurev-arplant-050312-120057 23394500

[B128] Sharrock K. R. Labavitch J. M. (1994). Polygalacturonase inhibitors of bartlett pear fruits differential effects on *Botrytis cinerea* polygalacturonase isozymes, and influence on products of fungal hydrolysis of pear cell walls and on ehylene induction in cell culture. *Physiol. Mol. Plant Pathol.* 45 305–319. 10.1016/S0885-5765(05)80061-X

[B129] Shi J. X. Adato A. Alkan N. He Y. H. Lashbrooke J. Matas A. J. (2013). The tomato SlSHINE3 transcription factor regulates fruit cuticle formation and epidermal patterning. *New Phytol.* 197 468–480. 10.1111/nph.12032 23205954

[B130] Simon-Plas F. Rusterucci C. Milat M. L. Humbert C. Montillet J. L. Blein J. P. (1997). Active oxygen species production in tobacco cells elicited by cryptogein. *Plant Cell Environ.* 20 1573–1579. 10.1046/j.1365-3040.1997.d01-45.x

[B131] Spoel S. H. Dong X. (2008). Making sense of hormone crosstalk during plant immune responses. *Cell Host Microbe* 3 348–351. 10.1016/j.chom.2008.05.009 18541211

[B132] Spoel S. H. Johnson J. S. Dong X. (2007). Regulation of tradeoffs between plant defenses against pathogens with different lifestyles. *Proc. Natl. Acad. Sci. U.S.A.* 104 18842–18847. 10.1073/pnas.0708139104 17998535 PMC2141864

[B133] Su H. Gubler W. D. (2012). Effect of 1-methylcyclopropene (1-MCP) on reducing postharvest decay in tomatoes (*Solanum lycopersicum* L.). *Postharvest Biol. Technol.* 64 133–137. 10.1016/j.postharvbio.2011.06.005

[B134] Sutton B. C. (1980). *The Coelomycetes. Fungi Imperfecti with Pycnidia, Acervuli and Stromata.* Kew: Commonwealth Mycological Institute.

[B135] Sutton B. C. (1992). “The genus *Glomerella* and its anamorph *Colletotrichum*,” in *Colletotrichum: Biology, Pathology and Control* eds Bailey J. A. Jeger J. M. (Wallingford: Centre for Biosciences and Agriculture International) 1–26.

[B136] Symons G. Chua Y.-J. Ross J. Quittenden L. Davies N. Reid J. (2012). Hormonal changes during non-climacteric ripening in strawberry. *J. Exp. Bot.* 63 4741–4750. 10.1093/jxb/ers147 22791823 PMC3428006

[B137] Symons G. Davies C. Shavrukov Y. Dry I. Reid J. Thomas M. (2006). Grapes on steroids. Brassinosteroids are involved in grape berry ripening. *Plant Physiol.* 150–158. 16361521 10.1104/pp.105.070706 PMC1326039

[B138] Tada Y. Spoel S. H. Pajerowska-Mukhtar K. Mou Z. Song J. Wang C. (2008). Plant immunity requires conformational charges of NPR1 via S-nitrosylation and thioredoxins. *Science* 321 952–956. 10.1126/science.1156970 18635760 PMC3833675

[B139] Tian S. Qin G. Li B. (2013). Reactive oxygen species involved in regulating fruit senescence and fungal pathogenicity. *Plant Mol. Biol.* 82 593–602. 10.1007/s11103-013-0035-2 23515879

[B140] Torres M. A. Jones J. D. Dangl J. L. (2006). Reactive oxygen species signaling in response to pathogens. *Plant Physiol.* 141 373–378. 10.1104/pp.106.079467 16760490 PMC1475467

[B141] van Loon L. C. Rep M. Pieterse C. M. J. (2006). Significance of inducible defense-related proteins in infected plants. *Annu. Rev. Phytopathol.* 44 135–162. 10.1146/annurev.phyto.44.070505.143425 16602946

[B142] vanKan J. A. L. vantKlooster J. W. Wagemakers C. A. M. Dees D. C. T. van der VlugtBergmans C. J. B. (1997). Cutinase a of *Botrytis cinerea* is expressed, but not essential, during penetration of gerbera and tomato. *Mol. Plant Microbe* 10 30–38. 10.1094/MPMI.1997.10.1.30 9002270

[B143] Verhoeff K. (1974). Latent infections by fungi. *Annu. Rev. Phytopathol.* 12 99–110. 10.1146/annurev.py.12.090174.000531

[B144] Voisin D. Nawrath C. Kurdyukov S. Franke R. B. Reina-Pinto J. J. Efremova N. (2009). Dissection of the complex phenotype in cuticular mutants of *Arabidopsis* reveals a role of SERRATE as a mediator. *PLoS Genet.* 5:e1000703 10.1371/journal.pgen.1000703 PMC2760142 19876373

[B145] Vorwerk S. Somerville S. Somerville C. (2004). The role of plant cell wall polysaccharide composition in disease resistance. *Trends Plant Sci.* 9 203–209. 10.1016/j.tplants.2004.02.005 15063871

[B146] Walton J. D. (1994). Deconstructing the cell wall. *Plant Physiol.* 104 1113–1118. 12232152 10.1104/pp.104.4.1113 PMC159271

[B147] Watkins C. B. (2006). The use of 1-methylcyclopropene (1-MCP) on fruits and vegetables. *Biotechnol. Adv.* 24 389–409. 10.1016/j.biotechadv.2006.01.005 16530376

[B148] Watkins C. B. Nock J. F. (2008). Effects of delayed controlled atmosphere storage of apples after rapid 1-MCP treatment. *Hortscience* 43 1087–1087.

[B149] Widmer T. L. Laurent N. (2006). Plant extracts containing caffeic acid and rosmarinic acid inhibit zoospore germination of *Phytophthora* spp. pathogenic to *Theobroma cacao*. *Eur. J. Plant Pathol.* 115 377–388. 10.1007/s10658-006-9024-5

[B150] Wildermuth M. C. (2006). Variations on a theme: synthesis and modification of plant benzoic acids. *Curr. Opin. Plant Biol.* 9 288–296. 10.1016/j.pbi.2006.03.006 16600669

[B151] Williams B. Kabbage M. Kim H. J. Britt R. Dickman M. B. (2011). Tipping the balance: *Sclerotinia sclerotiorum* secreted oxalic acid suppresses host defenses by manipulating the host redox environment. *PLoS Pathog.* 7:e1002107 10.1371/journal.ppat.1002107 PMC3128121 21738471

[B152] Williamson B. Tudzynsk B. Tudzynski P. van Kan J. A. L. (2007). *Botrytis cinerea*: the cause of grey mould disease. *Mol. Plant Pathol.* 8 561–580. 10.1111/j.1364-3703.2007.00417.x 20507522

[B153] Wojtaszek P. Trethowan J. Bolwell G. P. (1995). Specificity in the immobilization of cell-wall proteins in response to different elicitor molecules in suspension-cultured cells of french bean (*Phaseolus-vulgaris* L). *Plant Mol. Biol.* 28 1075–1087. 10.1007/BF00032668 7548825

[B154] Woloshuk C. P. Kolattukudy P. E. (1986). Mechanism by which contact with plant cuticle triggers cutinase gene-expression in the spores of *Fusarium-solani* F-Sp Pisi. *Proc. Natl. Acad. Sci. U.S.A.* 83 1704–1708. 10.1073/pnas.83.6.1704 16593666 PMC323152

[B155] Woolf A. B. Requejo-Tapia C. Cox K. A. Jackman R. C. Gunson A. Arpaia M. L. (2005). 1-MCP reduces physiological storage disorders of ‘Hass’ avocados. *Postharvest Biol. Technol.* 35 43–60. 10.1016/j.postharvbio.2004.07.009

[B156] Yakoby N. Kobiler I. Dinoor A. Prusky D. (2000). pH regulation of pectate lyase secretion modulates the attack of *Colletotrichum gloeosporioides* on avocado fruits. *Appl. Environ. Microbiol.* 66 1026–1030. 10.1128/AEM.66.3.1026-1030.2000 10698767 PMC91938

[B157] Zaharah S. S. Singh Z. Symons G. M. Reid J. B. (2012). Role of brassinosteroids, ethylene, abscisic acid, and indole-3-acetic acid in mango fruit ripening. *J. Plant Growth Regul.* 31 363–372. 10.1007/s00344-011-9245-5

[B158] Zhao J. Davis L. C. Verpoorte R. (2005). Elicitor signal transduction leading to production of plant secondary metabolites. *Biotechnol. Adv.* 23 283–333. 10.1016/j.biotechadv.2005.01.003 15848039

[B159] Zhu Z. Zhang Z. Q. Qin G. Z. Tian S. P. (2010). Effects of brassinosteroids on postharvest disease and senescence of jujube fruit in storage. *Postharvest Biol. Technol.* 56 50–55. 10.1016/j.postharvbio.2009.11.014

